# Types of second primary cancer influence overall survival in cutaneous melanoma

**DOI:** 10.1186/s12885-021-08845-x

**Published:** 2021-10-19

**Authors:** Guoqiao Zheng, Subhayan Chattopadhyay, Kristina Sundquist, Jan Sundquist, Asta Försti, Akseli Hemminki, Kari Hemminki

**Affiliations:** 1 Division of Molecular Genetic Epidemiology, German Cancer Research Center (DKFZ), Im Neuenheimer Feld 580, D-69120 Heidelberg, Germany; 2 Center for Primary Health Care Research, Lund University, 205 02 Malmö, Sweden; 3 Department of Family Medicine and Community Health, Department of Population Health Science and Policy, Icahn School of Medicine at Mount Sinai, New York, USA; 4 Center for Community-based Healthcare Research and Education (CoHRE), Department of Functional Pathology, School of Medicine, Shimane University, Matsue, Japan; 5 Hopp Children’s Cancer Center (KiTZ), 69120 Heidelberg, Germany; 6 Division of Pediatric Neurooncology, German Cancer Research Center (DKFZ), German Cancer Consortium (DKTK), Heidelberg, Germany; 7 Cancer Gene Therapy Group, Translational Immunology Research Program, University of Helsinki, Helsinki, Finland; 8 Comprehensive Cancer Center, Helsinki University Hospital, Helsinki, Finland; 9 Faculty of Medicine and Biomedical Center in Pilsen, Charles University in Prague, 30605 Pilsen, Czech Republic; 10 Division of Cancer Epidemiology, German Cancer Research Center (DKFZ), Im Neuenheimer Feld 580, D-69120 Heidelberg, Germany

**Keywords:** Independent primary, Second melanoma, Prognosis, Time-dependent analysis, Overall survival

## Abstract

**Background:**

Favorable survival in malignant cutaneous melanoma (melanoma) has increased the likelihood of second primary cancer (SPC). We assess the influence of patient characteristics at diagnosis of first melanoma and the type of SPC (second melanoma and other SPC) on overall survival.

**Methods:**

We used the Swedish Cancer Registry data to assess overall survival in melanoma for the period 1990 to 2015. Kaplan-Meier curves were plotted and hazard ratios (HRs) were estimated with Cox regression models by considering SPC diagnosis as a time-dependent variable.

**Results:**

A total of 46,726 patients were diagnosed with melanoma, and 15.3% of them developed SPC, among which, two thirds were other SPCs. Second melanomas were diagnosed early (31% during the first year) compared to non-melanoma SPCs (9.5%). Survival for women with second melanoma or other SPC (56 and 21% alive after 25 years of follow-up, respectively) exceeded the male rates (21 and 10%, respectively) but all these figures were lower than for females (60% alive) or males (48%) without SPC. Time dependent analysis showed vastly increased HRs for cancer types that are fatal also as first cancers, but SPC-specific HRs remained relatively uniform, irrespective of SPC diagnosed soon or late after first melanoma. In early-onset melanoma, SPC diagnosis after 10 years may not negatively influence overall survival.

**Conclusions:**

As the overall survival of patients with many types of SPCs is unfavorable, advice about health lifestyle should benefit smoking patients and early detection methods may be recommended for SPCs of the breast, prostate and colorectum.

**Supplementary Information:**

The online version contains supplementary material available at 10.1186/s12885-021-08845-x.

## Introduction

Survival rates in malignant cutaneous melanoma (subsequently ‘melanoma’) have improved in Europe and North America at the same time when there has been an increase in incidence [1–3]. In Sweden the 5-year relative survival for males with melanoma increased from 54.5% in 1960–1964 to 88% % in 1990–1999; the corresponding rates for females with melanoma were 65.8 and 94%, respectively [4, 5]. However, some of the latest data may indicate that the favorable trend in survival may have levelled off in USA and Sweden [2, 3]. Increasing survival implies that the likelihood of second primary cancers (SPCs) also increases. The increased risk for second or multiple melanomas in melanoma patients is well known [6–10]. However, non-melanoma SPCs are more common than second melanomas but they tend to be diagnosed later than second melanomas; our recent data showed that SPCs including second melanoma were diagnosed in 13.3% of melanoma patients [9]. That study also showed that SPCs interfere with overall favorable survival in melanoma.

Here we want to characterize the relationships for overall survival depending on gender, age at diagnosis and time interval between first melanoma and common types of SPCs using data from the Swedish Cancer Registry. The results have clinical implications.

## Methods

Patients diagnosed with histology-verified first primary invasive melanoma between 1990 and 2015 were identified in Swedish Cancer Registry according to International Classification of Diseases 7th revision (ICD-7) and they were followed for diagnosis of any of the 35 different SPCs including second melanomas. The follow-up for overall survival was started after first melanoma diagnosis and terminated at the date of emigration, death, or 31st December 2015, whichever came earliest.

The effect of time intervals from first melanoma to SPC diagnosis (no SPC, less than 1 year, 1–5 years, 6–10 years and longer than 10 years) and age at diagnosis of first melanoma (< 60, 60–79 and ≥ 80 years) were modeled as one variable with 15 levels in Cox proportional hazard model. Patients who were diagnosed before 60 years old and without SPC diagnosis were used as the reference group. In the model, SPC diagnosis was considered to be time-dependent in order to avoid the immortal time bias [11]. Patients were stratified based on their time from first melanoma to SPC; HRs were estimated according to diagnosis of SPC (time-dependent variable). Therefore, HRs are for survival from SPC diagnosis until follow-up end. The analyses were done separately for males and females and second melanoma and other SPC as well as some specific SPCs. The data fulfilled the proportional hazards assumption for the Cox model. In the model, year of diagnosis of first primary melanoma, place of residence (big cities, northern Sweden, southern Sweden and unspecific) and socio-economic status (blue-collar worker, white-collar worker, farmer, private business, professional, or other/unspecified) were additionally adjusted. We performed sensitivity analysis with the same method but stratification on the patients whose follow-up time was less than and over 6 years, thus setting the reference group homogeneous with the time to SPC. Kaplan-Meier curves were generated with stratification on time intervals from first melanoma to SPC diagnosis, age at diagnosis of first melanoma and gender.

Statistical analyses were done with R version 3.4, SAS version 9.4 and Stata version 15.1.

## Results

Characteristics of the melanoma cohort are described in Table 1. A total of 46,726 patients were diagnosed with melanoma, including marginally more females (51.1%). Case numbers showed a vast increase over the 5-year periods (note the first period was 6 years). SPCs were developed in 15.3% of the patients, among which 1/3 were second melanomas and 2/3 other SPCs. Second melanomas were diagnosed early (31% during the first year) compared to other SPCs (9.5%). Additional data, not shown in Table 1: median (interquartile range, IQR) time from first melanoma to SPC was 4 (1–9), 2 (0–7) and 5 (2–10) years for any SPCs, second melanoma and other SPC, respectively. Median (IQR) age at diagnosis of first melanoma was 66 (56–74), 63 (51.5–73) and 66 (58–7​5) years for patients with any SPCs, second melanoma and other SPC, respectively. Median (IQR) age at diagnosis of SPC was correspondingly 72 years (63–80), 68 (57–77) and 73 (65–81) years.

**Table 1:** Characteristics in melanoma patients diagnosed during 1990 to  2015

Covariate			Number of cases	Percentage (%)
**Age at diagnosis (years old)**		< 60	21,164	45.3
		60–79	19,259	41.2
		≥ 80	6303	13.5
**Gender**		Male	22,867	48.9
		Female	23,859	51.1
**Year at diagnosis**		1990–1995	7824	16.7
		1996–2000	6888	14.7
		2001–2005	8224	17.6
		2006–2010	10,335	22.1
		2011–2015	13,455	28.8
**SPC diagnosis**		No SPC	39,589	84.7
		Second melanoma	1856	4.0
		Other SPC	5281	11.3
**Interval from first melanoma to SPC (yrs)**	SPC=Second melanoma	< 1	575	31.0
		1–5	635	34.3
		6–10	305	16.4
		> 10	340	18.3
	SPC=Other SPC	< 1	500	9.5
		1–5	1999	37.9
		6–10	1402	26.6
		> 10	1380	26.1

After 25 years follow-up, 60% of females and 48% of males survived. The difference of overall survival between genders was even higher for patients with second melanoma (56% vs. 21%) but less for those with other SPCs (19% vs.10%). Table 2 displays HRs for overall survival in time-dependent analysis using multivariable Cox regression. Male and female overall survival data after second melanoma are shown on the top part of Table 2. Advanced age at diagnosis of first melanoma was associated with increased HRs irrespective of SPC diagnoses. In age groups with many deaths (60–79 years) HRs for overall survival were quite constant, approximately 3.0–4.0 for males and 5.0–6.0 for females, irrespective of the time interval from first to second cancer. When SPC was non-melanoma (lower part of Table 2) the survival trends were similar to patients with second melanoma, except that the HRs were clearly higher. For males and females diagnosed between ages 60–79 years, HRs were between 7.0–8.0 for males and 11–15 for females; for those diagnosed at age over 79 years, the corresponding HRs range were 10–14 and 16–22. In addition to the numbers of deaths shown in Table 2, we show the numbers of underlying person-years in Supplementary Table 1. The results were similar for stratification on the patients whose follow-up time was less than and over 6 years (Supplementary Table 2). Although among patients whose follow-up time equal or longer than 6 years, the effect of SPCs on overall survival was stronger than those with follow-up time less than 6 years.

**Table 2:** Hazard ratio of overall survival in melanoma patients based on gender, age at diagnosis of melanoma and interval from first melanoma to SPC diagnosis

Gender	Age at diagnosis (years old)	No SPC		Interval from first melanoma diagnosis to SPC							
				< 1 year		1–5 year		6–10 year		> 10 year	
		N	HR (95% CI)	N	HR (95% CI)	N	HR (95% CI)	N	HR (95% CI)	N	HR (95% CI)
**Second melanoma**											
Males	< 60	1304	Reference	20	2.41 (1.54–3.77)	36	2.81 (2.00–3.94)	10	0.87 (0.47–1.63)	13	0.63 (0.36–1.11)
	60–79	3189	2.96 (2.76–3.16)	59	3.98 (3.12–5.07)	72	4.39 (3.47–5.56)	48	4.75 (3.64–6.19)	27	4.76 (3.33–6.80)
	≥80	1677	8.17 (7.53–8.86)	30	7.57 (5.41–10.6)	30	11.1 (7.90–15.6)	8	7.16 (4.57–11.2)		
Females	< 60	840	Reference	6	1.37 (0.59–3.18)	14	1.64 (0.94–2.87)	3	0.42 (0.14–1.29)	5	0.28 (0.12–0.68)
	60–79	2062	4.23 (3.89–4.59)	24	5.09 (3.39–7.63)	33	6.19 (4.34–8.84)	17	5.92 (3.66–9.57)	16	6.08 (3.85–9.60)
	≥80	1964	14.3 (13.1–15.7)	23	15.9 (10.6–23.9)	25	17.2 (12.0–24.5)	9	19.9 (11.1–35.8)	2	22.4 (9.66–52.1)
**Other SPC**											
Males	< 60	1304	Reference	15	5.16 (2.95–9.02)	59	3.71 (2.77–4.97)	65	3.61 (2.79–4.67)	100	2.90 (2.30–3.66)
	60–79	3189	2.96 (2.77–3.17)	108	7.65 (6.21–9.43)	443	7.20 (6.42–8.08)	309	7.40 (6.52–8.39)	201	8.18 (7.03–9.51)
	≥80	1677	8.29 (7.65–8.98)	57	13.5 (10.7–17.1)	174	11.9 (10.2–13.9)	44	14.2 (10.6–19.1)	3	10.0 (2.80–35.9)
Females	< 60	840	Reference	15	4.89 (2.77–8.64)	66	5.97 (4.5–7.91)	65	4.56 (3.47–6.00)	104	3.81 (3.04–4.77)
	60–79	2062	4.25 (3.91–4.61)	51	13.0 (9.26–18.4)	213	11.7 (9.9–13.7)	181	11.9 (9.94–14.3)	125	15.3 (12.3–19.1)
	≥80	1964	14.5 (13.2–15.8)	36	17.6 (12.2–25.3)	136	22.5 (18.8–27.1)	40	16.4 (11.8–23.0)	8	17.4 (8.14–37.4)

*N* number of death, *HR* hazard ratio, *CI* confidence interval, *SPC* second primary cancer

**Fig. 1:**
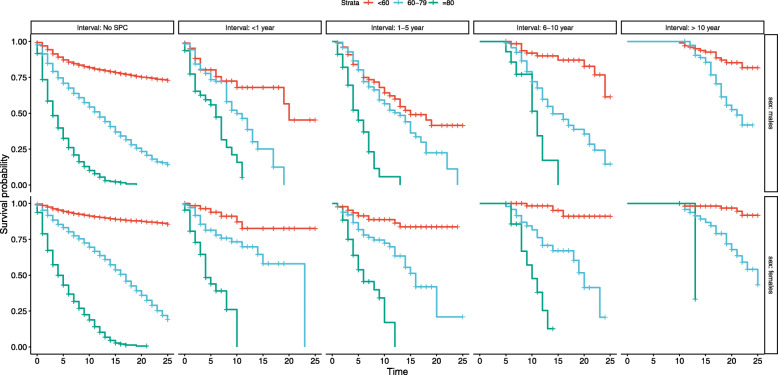
Survival probability stratified by time intervals from first melanoma to second melanoma diagnosis, gender and age at diagnosis of first melanoma in melanoma patients with/without second melanoma diagnosis. Strata, age at diagnosis of first melanoma. Interval: time intervals from first melanoma to second melanoma diagnosis. Time, survival time from first melanoma diagnosis (in years)

**Fig. 2:**
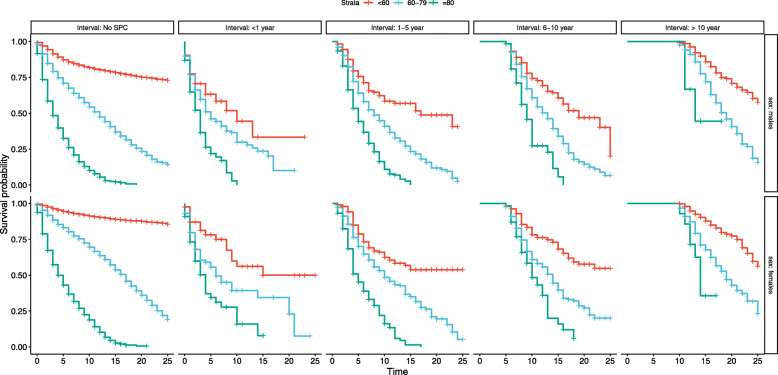
Survival probability stratified by time intervals from first melanoma to SPC diagnosis, gender and age at diagnosis of first melanoma in melanoma patients with/without other SPC diagnosis. Interval: time intervals from first melanoma to SPC diagnosis. Strata, age at diagnosis of first melanoma. Time, survival time from first melanoma diagnosis (in years). SPC, second primary cancer other than melanoma

Kaplan-Meier curves are shown in Figs. 1 to 2 stratified by gender and type of SPC (melanoma, other), age at diagnosis of first melanoma and time interval between melanoma and SPC. As one of the stratified variables was the time interval from first melanoma to SPC there were no deaths before the allowed follow-up started; this is shown as a straight line with 100% survival. Other features were the generally better overall survival for young compared to old patients, patients with second melanoma compared to those with other SPC and females compared to males specifically when first melanoma was diagnosed before 60 and SPC was melanoma. The decline in overall survival became ever steeper the longer the interval to SPC was.

In Table 3 we show overall survival for common individual cancers as in Table 2. The different cancers were ordered based on the known survival from the best (skin) to the worst (lung and cancer of unknown primary, CUP). HRs followed approximately this order in the various interval groups. Again HRs over the age and interval groups were relatively constant for the particular cancers. A minor exception was second CUP diagnosed six or more years after melanoma: HRs were 50.0 or more. Of note, for any of the listed SPCs, the HRs were below 1.0 when the first melanoma was diagnosed before age 60 and when more than 10 years elapsed from first melanoma to diagnosis of SPC except for lung cancer.

**Table 3:** Hazard ratio of overall survival in melanoma patients based on age at diagnosis of melanoma and interval from first melanoma to specific SPC diagnosis

Age at diagnosis (years old)	No SPC		Interval to SPC from first melanoma diagnosis							
			< 1 year		1–5 year		6–10 year		> 10 year	
	N	HR (95% CI)	N	HR (95% CI)	N	HR (95% CI)	N	HR (95% CI)	N	HR (95% CI)
**Squamous cell skin cancer**										
< 60	2144	Reference	2	2.1 (0.6–6.5)	6	1.7 (0. 8–3.7)	8	1.0 (0.52–2.0)	7	0.2 (0.1–0.5)
60–79	5251	3.9 (3.7–4.1)	22	6.7 (4.5–10.0)	75	5.7 (4. 6–7.1)	62	6.5 (5.0–8.3)	50	6.8 (5.1–9.1)
≥ 80	3641	15.7 (14.8–16.6)	42	20.0 (14.9–26.7)	91	17.7 (14.8–21.2)	24	16.1 (11.4–22.6)	6	19.6 (10.0–38.5)
**Breast cancer**										
< 60	2144	Reference	4	1.8 (0.6–5.1)	18	1.9 (1.2–3.0)	17	1.3 (0.8–2.0)	19	0.5 (0.3–0.8)
60–79	5251	3.9 (3.7–4.1)	12	9.1 (5.8–14.2)	36	5.6 (4.0–7.7)	25	6.2 (4.3–8.9)	16	7.4 (4.6–12.0)
≥ 80	3641	15.7 (14.8–16.6)	7	17.5 (9.3–33.1)	24	23.4 (16.6–32.9)	12	18.1 (10.7–30.6)	2	25.6 (3.07–212.4)
**Prostate cancer**										
< 60	2144	Ref	1	1.2 (0.2–9.0)	9	1.2 (0.6–2.4)	10	0.8 (0.4–1.5)	14	0.4 (0.2–0.6)
60–79	5251	3.9 (3.7–4.1)	22	5.9 (4.0–8.8)	139	6.4 (5.4–7.6)	96	5.9 (4.9–7.1)	65	7.6 (6.1–9.5)
≥ 80	3641	15.8 (14.9–16.7)	7	16.7 (9.3–30.0)	46	17.2 (14.0–21.1)	13	29.3 (17.0–50.5)	1	29.5 (27.4–31.7)
**Colorectal cancer**										
< 60	2144	Reference	2	2.8 (0.5–14.8)	10	2.9 (1.6–5.3)	16	2.1 (1.3–3.4)	22	0.7 (0.4–1.0)
60–79	5251	3.9 (3.7–4.1)	24	13.3 (8.3–21.5)	62	11.3 (9.0–14.3)	68	13.1 (10.2–16.8)	39	10.4 (7.6–14.3)
≥ 80	3641	15.7 (14.8–16.6)	11	17.4 (10.2–29.6)	46	26.5 (18.6–37.7)	9	28.5 (16.0–50.9)	0	0 (0–0)
**Non-Hodgkin lymphoma**										
< 60	2144	Reference	4	4.7 (2.4–9.4)	7	1.9 (0.9–4.1)	1	0.2 (0.0–1.1)	7	0.2 (0.1–0.5)
60–79	5251	3.9 (3.7–4.1)	7	21.5 (9. 9–46.7)	42	13.9 (10.7–18.1)	18	15.7 (9.1–27.2)	14	13.4 (7.2–24.7)
≥ 80	3641	15.67 (14.8–16.6)	1	50.0 (45.9–54.6)	12	27.6 (14.6–51.8)	0		0	
**Lung cancer**										
< 60	2144	Reference	5	15.3 (6.8–34.5)	11	3.7 (2.0–6.9)	15	2.4 (1.4–4.0)	32	1.1 (0.8–1.6)
60–79	5251	3.9 (3.7–4.1)	16	28.7 (16.1–51.2)	64	42.3 (29.3–61.0)	34	35.2 (21.9–56.5)	31	34.6 (25.1–47.6)
≥ 80	3641	15.7 (14.8–16.6)	3	56.9 (23.6–136.9)	7	62.5 (36.5–106.9)	6	–	1	–
**Cancer of unknown primary**										
< 60	2144	Reference	1	1.7 (0.2–14.2)	13	4.9 (2.8–8.6)	13	2.2 (1.2–3.7)	22	0.8 (0.5–1.2)
60–79	5251	3.9 (3.7–4.1)	6	40.0 (13.7–116.8)	28	35.0 (17.6–69.7)	22	50.0 (32.4–77.1)	22	97.0 (66.3–141.9)
≥ 80	3641	15.7 (14.8–16.6)	3	23.8 (9.71–58.2)	13	50.2 (16.2–155.7)	1	231.6 (210.3–255.1)	1	227.0 (206.2–249.8)

*N* number of death, *HR* hazard ratio, *CI* confidence interval, *SPC* second primary cancer

## Discussion

Therapy for melanoma has experienced a revolution as a result of targeted therapies with BRAF and MEK inhibitors and immunotherapies with checkpoint inhibitors [12, 13]. However, as these therapies were introduced in Europe in the mid-2010s, their possible influence would not be shown in the present population-based figures. Data from the Stockholm Melanoma Register showed that 98.9% of all patients underwent surgery as the first-line treatment, and thus therapeutic side-effects are unlikely to influence the current survival estimates [14]. Many survival studies on cancer, including melanoma, focus on disease-specific survival, which may be different from overall survival, particularly for cancers of good survival, such as melanoma. For treatment-specific effects, disease-specific survival is of main interest but for the study of co-morbidities (such as SPCs), overall survival is of primary interest. Thus SPCs as cause of mortality in melanoma have largely been overlooked. According to an Australian study based on 20-year survival in thin melanoma (1 mm or less, accounting for 71% of all) only 2.2% of the patients had died of melanoma [15]. In a Swedish study covering years 1958 through 2015 and median follow-up time since melanoma of 8 years, 74.2% of patients without SPC died of melanoma while for those diagnosed with SPC, first melanoma was the cause of death only among 24.5% of the patients; 43.1% died of SPC other than melanoma [9].

Age at diagnosis is known to influence negatively both melanoma-specific and overall survival and we showed here that it also negatively influences overall survival in patients with SPC [1, 16]. Similarly, females survive better in primary melanoma than males, and we showed here that the difference was also seen in SPCs after melanoma, including second melanoma [1, 2, 16]. The survival advantage in women may be related to known enhanced immunoreactivity in women which is manifested in the overall excess of autoimmune diseases in women compared to men; it is believed to be related at least in part to estrogen modulation of immune functions [17, 18].

The current data on overall survival in melanoma patients, focusing on the time interval from first melanoma to SPCs, revealed two novel features. One with practical clinical implications suggests that in young melanoma patients, diagnosed before age 60, second melanomas may not decrease survival; however, case numbers were few. This applied even to melanomas diagnosed before age 60 with development of SPCs in skin, breast and prostate cancers after more than 10 years of melanoma diagnosis; this was even true when diagnosis was 6 or more years after melanoma. However, the numbers of deaths in these age groups were small, thus warranting caution. The possible explanation could be an increased awareness of patients to detect early signs of some cancers following the initial melanoma diagnosis. However, the information on cancer screening and clinical staging is not available in the current study.

The second novel feature was that, overall, the HRs were relatively uniform over the time intervals for diagnostic age- and SPC-specific overall survival. This appears to be contradictory to the Kaplan-Meier data shown in Figs. 1 to 2, which described an ever steeper decline in overall survival when the interval time was extended. However, the HRs were calculated using time-dependent Cox proportional hazard regression analysis which adjusted for the interval-defined waiting time (referred to as for ‘immortal time’) when no deaths occur by definition [11]. In this model the time interval to diagnosis of SPC is treated as a time-dependent variable to avoid the immortal time bias [11]. Time-dependent data describe the true clinical situation, i.e., the overall survival for melanoma patients diagnosed with SPC was fairly constant whether SPC was diagnosed shortly after the first diagnosis or 10 years later. The only exception was for relatively young patients, as discussed in the previous paragraph.

We showed also for a number of specific cancers as SPC that those known to be fatal as first cancer were also fatal as SPC. Similar results have been reported for second cancers after hematological malignancies [19, 20]. However, even for cancers with moderate overall survival as first cancers, such as colorectal cancer and Hodgkin lymphoma, HRs as SPC were as high as around 13 and 15, respectively, when melanoma was diagnosed at age 60 to 79 years; in melanoma patients without SPC the background HR was 3.87. In melanoma patients in that age group who were unfortunate to be diagnosed with second lung cancer or CUP, HRs ranged from 30 to about 100 which is practically a death sentence. CUP is diagnosed as metastases and it is highly fatal also as first cancer [21–23].

The strengths of the present study include practically complete coverage of histologically verified cancers diagnosed in Sweden and a complete coverage of deaths. The potential weaknesses are related to epidemiology of melanoma with vastly increasing incidence and improved survival which may introduce inaccuracies in the calculations. Although the law mandates reporting all cancers to the cancer registry there is only a small study verifying a high rate of reporting of SPCs [24].

In conclusion, survival analysis on melanoma patients with SPCs showed a lower overall survival in patients with second melanoma and in particular those with other SPCs. The overall survival in SPC followed the expected overall survival from first cancer, patients with second lung cancer and CUP showed a very poor overall survival. For patients with SPC, a female overall survival advantage was noted over males, in addition to a strongly worsening overall survival in older patients, both in line with survival data in primary melanoma. Time-dependent analysis suggested that young melanoma patients diagnosed with SPC 6 or 10 years later may not have worse overall survival. It further showed that for melanoma diagnosed at common age groups, overall survival was relatively constant in patients with particular SPCs irrespective of the time interval between first melanoma and SPC. As the outcome of patients with many types of SPC is unfavorable, the cancer prevention and early detection should be considered for melanoma patients. For the most fatal SPCs of the lung and unknown primary smoking is an important risk factor and advice about smoking cessation may be particularly well received at diagnosis of primary cancer [25]. For the other common SPCs of the breast, prostate and colorectum screening methods are available and could be recommended at least for elderly patients with the highest risk.

## Supplementary Information

**Additional file 1.**

## Data Availability

The data that support the findings of this study are available from Lund University but restrictions apply to the availability of these data, which were used under license for the current study and so are not publicly available. Any request regarding the data from this study should go to the last author (K.H.).
