# Supplementary material for: Types of second primary cancer influence overall survival in cutaneous melanoma
Source: BMC Cancer. 2021 Oct 19;21:1123. doi: 10.1186/s12885-021-08845-x (PMC8524825; doi:10.1186/s12885-021-08845-x)
Supplement: Supplementary file 1 — Additional file 1. [file 12885_2021_8845_MOESM1_ESM.docx]

Supplementary table 1 Person-years at risk in melanoma patients based on gender, age at diagnosis and interval from first melanoma to SPC diagnosis

| Gender | Age at diagnosis (years old) | No SPC | | Interval to SPC from first melanoma diagnosis | | | | | | | |
| --- | --- | --- | --- | --- | --- | --- | --- | --- | --- | --- | --- |
|  |  |  |  | <1 year | | 1-5 year | | 6-10 year | | > 10 year | |
|  |  | N | Pyrs | N | Pyrs | N | Pyrs | N | Pyrs | N | Pyrs |
| **Second melanoma** | | | | | | | | | | | |
| Males | <60 | 1304 | 71338 | 20 | 478 | 36 | 873 | 10 | 971 | 13 | 1835 |
|  | 60-79 | 3189 | 47792 | 59 | 836 | 72 | 1333 | 48 | 1157 | 27 | 1297 |
|  | ≥80 | 1677 | 7534 | 30 | 179 | 30 | 186 | 8 | 121 |  |  |
|  | <60 | 840 | 101815 | 6 | 518 | 14 | 1164 | 3 | 923 | 5 | 2035 |
|  | 60-79 | 2062 | 49398 | 24 | 647 | 33 | 838 | 17 | 602 | 16 | 892 |
|  | ≥80 | 1964 | 11482 | 23 | 147 | 25 | 209 | 9 | 133 | 2 | 49 |
| **Other SPC** | | | | | | | | | | | |
|  | <60 | 1304 | 71338 | 15 | 176 | 59 | 1322 | 65 | 2137 | 100 | 6471 |
|  | 60-79 | 3189 | 47792 | 108 | 802 | 443 | 5194 | 309 | 5708 | 201 | 6164 |
|  | ≥80 | 1677 | 7534 | 57 | 187 | 174 | 1090 | 44 | 543 | 3 | 79 |
|  | <60 | 840 | 101815 | 15 | 364 | 66 | 1763 | 65 | 2784 | 104 | 7234 |
|  | 60-79 | 2062 | 49398 | 51 | 464 | 213 | 3090 | 181 | 3791 | 125 | 4021 |
|  | ≥80 | 1964 | 11482 | 36 | 189 | 136 | 906 | 40 | 581 | 8 | 186 |

N, number of death, SPC, second primary cancer, pyrs, person-years at risk

Supplementary table 2 Hazard ratio of overall survival in melanoma patients stratified by total follow-up (diagnosis of melanoma to the stop of the follow-up).

| Individuals whose total follow-up time | Type of SPC | Gender | Age at diagnosis (years old) | **No SPC** | | Interval to SPC from first melanoma diagnosis | | | |
| --- | --- | --- | --- | --- | --- | --- | --- | --- | --- |
|  |  |  |  |  |  | <6 year | | ≥6 year | |
|  |  |  |  | N | HR (95% CI) | N | HR (95% CI) | N | HR (95% CI) |
| <6 year | Second melanoma | Males | <60 | 756 | Reference | 29 | 1.09(0.85-1.4) | - | - |
|  |  |  | 60-79 | 1757 | 1.24(1.14-1.34) | 56 | 1.25(1-1.57) | - | - |
|  |  |  | ≥80 | 1243 | 1.69(1.54-1.86) | 38 | 1.91(1.37-2.65) | - | - |
|  |  | Females | <60 | 434 | Ref | 10 | 0.9(0.53-1.53) | - | - |
|  |  |  | 60-79 | 856 | 1.48(1.31-1.67) | 30 | 1.91(1.37-2.67) | - | - |
|  |  |  | ≥80 | 1281 | 2.08(1.83-2.35) | 33 | 1.42(1.04-1.94) | - | - |
|  | Other SPC | Males | <60 | 756 | Reference | 43 | 1.82(1.43-2.32) | - | - |
|  |  |  | 60-79 | 1757 | 1.24(1.14-1.35) | 289 | 1.83(1.59-2.1) | - | - |
|  |  |  | ≥80 | 1243 | 1.72(1.56-1.88) | 145 | 2.06(1.74-2.44) | - | - |
|  |  | Females | <60 | 434 | Reference | 35 | 1.61(1.14-2.29) | - | - |
|  |  |  | 60-79 | 856 | 1.48(1.31-1.68) | 132 | 2.76(2.24-3.4) | - | - |
|  |  |  | ≥80 | 1281 | 2.08(1.83-2.35) | 116 | 1.95(1.53-2.48) | - | - |
| ≥6 year | Second melanoma | Males | <60 | 548 | Reference | 27 | 3.08(2.05-4.63) | 21 | 1.00(0.65-1.54) |
|  |  |  | 60-79 | 1432 | 4.20(3.78-4.67) | 75 | 6.76(5.29-8.65) | 49 | 5.49(4.18-7.21) |
|  |  |  | ≥80 | 434 | 12.2(10.6-14.1) | 22 | 14.0(9.90-19.8) | 11 | 12.4 (7.37-20.8) |
|  |  | Females | <40 | 406 | Ref | 10 | 1.55(0.79-3.05) | 8 | 0.51(0.26-1.04) |
|  |  |  | 60-79 | 1206 | 6.03(5.36-6.78) | 27 | 6.10(4.18-8.88) | 33 | 6.63(4.53-9.70) |
|  |  |  | ≥80 | 683 | 21.0(18.2-24.2) | 15 | 21.1(14.7-30.4) | 16 | 36.2(20.8-63.0) |
|  | Other SPC | Males | <60 | 548 | Reference | 31 | 3.77(2.53-5.62) | 165 | 4.07(3.34-4.96) |
|  |  |  | 60-79 | 1432 | 4.14(3.73-4.60) | 262 | 9.07(7.75-10.6) | 510 | 10.1(8.82-11.5) |
|  |  |  | ≥80 | 434 | 11.8 (10.4-13.6) | 86 | 15.7(13.0-18.9) | 47 | 25.0(17.6-35.6) |
|  |  | Females | <60 | 406 | Reference | 46 | 6.49(4.54-9.30) | 169 | 5.64(4.63-6.88) |
|  |  |  | 60-79 | 1206 | 6.03(5.37-6.77) | 132 | 12.7(10.4-15.6) | 306 | 17.2(14.4-20.5) |
|  |  |  | ≥80 | 683 | 20.2(17.5-23.0) | 56 | 21.7(17.1-27.5) | 48 | 26.0 (18.1-37.4) |

N, number of death, HR, hazard ratio, CI, confidence interval, SPC, second primary cancer
